# Primary abdominal lymphangioleiomyomatosis: report of a case

**DOI:** 10.1186/s12957-015-0512-y

**Published:** 2015-03-05

**Authors:** Yuan Ding, Sheng Yan, Yang Tian, Zhiwei Li, Jun Pan, Qiyi Zhang, Yan Wang, Shusen Zheng

**Affiliations:** Division of Hepatobiliary and Pancreatic Surgery, First Affiliated Hospital, School of Medicine, Zhejiang University, 79# QingChun Road, Hangzhou, 310003 People’s Republic of China; Key Laboratory of Combined Multi-organ Transplantation, Ministry of Public Health, The First Affiliated Hospital, School of Medicine, Zhejiang University, 79# QingChun Road, Hangzhou, 310003 People’s Republic of China; Key Laboratory of Organ Transplantation Zhejiang Province, First Affiliated Hospital, School of Medicine, Zhejiang University, 79# QingChun Road, Hangzhou, 310003 China

**Keywords:** Lymphangioleiomyomatosis, Extrapulmonary, Smooth muscle cells, Immunohistochemistry, CD117

## Abstract

Lymphangioleiomyomatosis is an uncommon progressive disease characterized by hamartomatous smooth muscle proliferation of the airways within the lungs as well as the lymph nodes, lymphatics, and blood vessels of the lungs, mediastinum, and abdomen. The most common manifestations of lymphangioleiomyomatosis are pulmonary symptoms. Primary abdominal lymphangioleiomyomatosis without any pathological changes in the respiratory system is extremely unusual. We report a case of primary abdominal lymphangioleiomyomatosis located between the left hepatic and gastric antrum of a 29-year-old woman. The patient had no typical symptoms of lymphangioleiomyomatosis (dyspnea, pneumothorax) or abdominal pain. All physical examination findings were normal. Laboratory test results, including routine blood examination, liver and kidney function, tumor markers, blood coagulation function, and urine and stool examinations, were all normal. She found abdominal cyst in an annual medical examination by ultrasonography and confirmed by computed tomography. For a clear diagnosis, a laparoscopic abdominal mass resection was performed. The postoperative pathohistological examination findings allowed for the definitive diagnosis. This case report may advance the understanding of primary peritoneal lymphatic leiomyoma and reduce the number of mistakenly diagnosed patients.

## Background

Lymphangioleiomyomatosis (LAM) was first reported 50 years ago; however, only about 100 cases have been published since then [1]. LAM is characterized by the proliferation of abnormal smooth muscle cells (LAM cells), which leads to the development of cystic lung lesions and lymphatic abnormalities [2]. Almost all cases of LAM in women occur during the reproductive period [3]. Microscopically, LAM is characterized by diffuse proliferation of smooth muscle cells (LAM cells) arranged in fascicular, trabecular, and papillary patterns associated with slit-like vascular channels. Immunohistochemical studies have shown strong reactivity of most LAM cells to α-smooth muscle actin and smooth muscle myosin heavy chain and weak to moderate reactivity of fewer cells to desmin and nonmuscle myosin heavy chain II-B [4].

According to previous reports, the initial symptoms and radiographic changes in patients with LAM are always associated with the respiratory system [5]. The most common manifestations of LAM are pulmonary symptoms including progressive dyspnea, recurrent pneumothoraces, and chylous effusions [6]. Computed tomography (CT) scans, the findings of which are almost pathognomonic, show numerous thin-walled cysts throughout the lungs [7].

Although it is reported this disease almost primarily affects the lungs, severe cases of extrapulmonary LAM have also been reported. As Matsui *et al.* described, many of patients with extrapulmonary LAM would develop pulmonary LAM within 1 to 2 years after diagnosis [3]. Common extrapulmonary presentations include retroperitoneal adenopathy (77%) and renal angiomyolipomas (60%), which can always be identified by CT scans as the disease progresses [7,8]. To the best of our knowledge, only four case reports of patients with LAM who presented with abdominal pain as the first symptom have been reported. However, pathological changes in the respiratory system were found in all of these patients after further examination [8]. The diagnosis of LAM almost completely depends on progressive respiratory symptoms and typical pulmonary imaging findings [9].

In summary, primary abdominal LAM (PA-LAM) is extremely difficult to diagnose because it shows no typical respiratory system symptoms or imaging changes [10]. LAM is considered to be an invasive disease, and a delay in the diagnosis may lead to a poor prognosis [11,12]. Therefore, we believe that patients with PA-LAM may benefit from early diagnosis, particularly by postoperative pathological examination, and intervention, including surgical resection. When a diagnosis of LAM is temporarily impossible, timely surgical treatment may be helpful for an early diagnosis, thereby improving the prognosis.

We herein describe a patient with PA-LAM with no respiratory or abdominal symptoms and an absence of any pulmonary imaging findings during a 6-month follow-up period. The diagnosis of PA-LAM was not made until a pathohistological examination was performed after surgical resection.

## Case presentation

A 27-year-old asymptomatic woman was admitted to the hospital because of an abdominal mass that had been detected during a routine examination. She denied having any respiratory symptoms, including cough, expectoration, asthma, fever, or dyspnea on exertion. Additionally, she had no history of abdominal pain, early satiety, flatulence, gastrointestinal bleeding, or vomiting throughout the last year. All physical examination findings were normal. Laboratory test results, including routine blood examination, liver and kidney function, tumor markers, blood coagulation function, and urine and stool examinations, were all normal.

**Figure 1:**
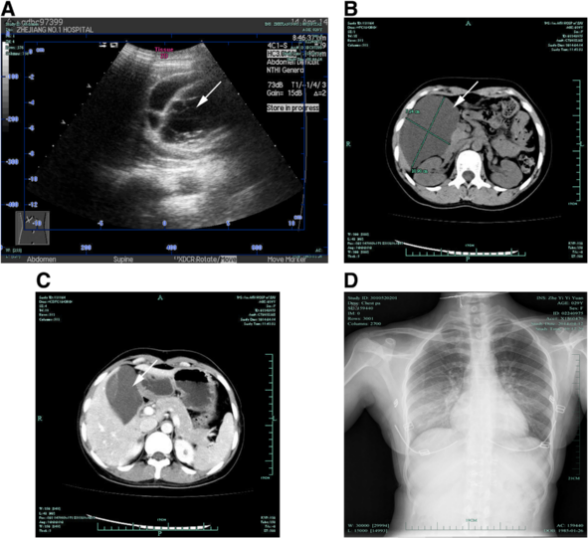
**Image findings.** Abdominal ultrasonography (US) showed a right abdominal cystic mass with segmentation, and the presence of a lymphatic cyst was considered **(A)**. An abdominal contrast-enhanced CT scan revealed a large cyst between the liver and stomach, approximately 10.47 × 66.10 cm in size **(B)**. The density of the cyst was homogeneous, and no obvious contrast signal was noted **(C)**. Chest X-ray showed no significant abnormalities before the operation **(D)**.

Abdominal ultrasonography (US) showed a right abdominal cystic mass with segmentation, and the presence of a lymphatic cyst was considered (Figure 1A). An abdominal contrast-enhanced CT scan revealed a large cyst between the liver and stomach, approximately 10.47 × 6.61 cm in size (Figure 1B). The density of the cyst was homogeneous, and no obvious contrast signal was noted (Figure 1C).

**Figure 2:**
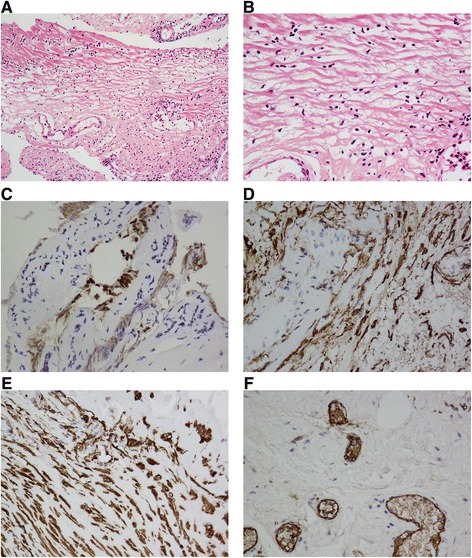
**H&E and immunohistochemical studies.** H&E showed the mass was filled with clear cystic fluid containing a small amount of a jelly-like substance. The final pathological examination showed that the cyst comprised various lymph vessels, and smooth muscle cells were observed in part of the capsule wall (**(A)** × 100 field, **(B)** × 400 field). Immunohistochemistry showed strong reactivity of most cells for D2-40 **(C)** and CD34 **(D)** and weak to moderate reactivity of fewer cells to smooth muscle actin **(E)** and F8-R-Ag **(F)**.

A chest X-ray showed no significant abnormalities before the operation (Figure 1D). A laparoscopic abdominal mass resection was performed to establish the patient’s diagnosis. At the time of the operation, a cystic mass about 10 cm in diameter was found near the hepatorenal ligament in the right abdomen. No metastatic lesions were detected in other organs. The cyst was gradually freed and completely resected. The duration of the operation was about 2 h, and the volume of blood loss was about 10 mL. On gross evaluation, the mass was filled with clear cystic fluid containing a small amount of a jelly-like substance. The final pathological examination showed that the cyst comprised various lymph vessels, and smooth muscle cells were observed in part of the capsule wall (Figure 2A). Immunohistochemical studies showed strong reactivity of most cells for D2-40 and CD34 and weak to moderate reactivity of fewer cells to smooth muscle actin and F8-R-Ag (Figure 2B).

In summary, the pathological findings indicated the presence of PA-LAM. According to previous reports, pulmonary LAM was found in almost all cases of extrapulmonary LAM during subsequent treatments. However, neither the preoperative chest X-ray nor the postoperative lung CT scan suggested the presence of pulmonary LAM.

**Figure 3:**
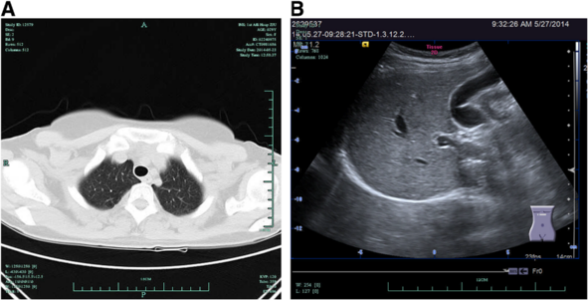
**Lung imaging and follow-up examination.** Lung CT scan **(A)** and abdominal ultrasonography **(B)** showed no obvious abnormality during the 1-year follow-up.

No adjuvant therapy, such as chemotherapy, was administered after the surgery. The patient recovered well and has had no evidence of recurrence. No pulmonary or abdominal abnormalities were found by CT scan (Figure 3A,B) during the 1-year follow-up.

## Discussion

LAM, a rare progressive disease of unknown etiology, is almost exclusively seen in women, normally in their reproductive years and at an average age of 41 years at diagnosis. The clinical presentation of LAM was first reported by Stossel in 1937. The typical pathological characteristics of LAM are hamartomatous proliferation of immature smooth muscle cells within the lymph nodes, lymphatics, blood vessels, and small airways. LAM primarily affects the lungs and mediastinum. Proliferation of LAM cells could result in the obstruction of the lymphatics, vessels, and airways, leading to the appearance of classic clinical symptoms such as chylous effusion, progressive dyspnea, recurrent pneumothorax, and hemoptysis. Jonson and Urban reported that the estimated prevalence of LAM is around 1 per 1 million women in the United Kingdom, France, and the United States [13,14]. While in Asia, however, Ling Ye performed a clinical analysis and reported an incidence of 2.6 per 1 million women without evidence of genetic disease [15].

The precise pathogenesis of LAM is unclear. One hypothesis states that smooth muscle proliferation is likely to be stimulated by steroidal hormones because the onset of LAM predominately occurs in premenopausal women, and exacerbation of LAM occurs during pregnancy or administration of estrogen [16]. Genetic studies have demonstrated that sporadic LAM is strongly related to a mutation of one of the tuberous sclerosis complex genes on chromosome 16 [17]. However, the exact mechanism of LAM remains to be elucidated.

Historically, the prognosis of LAM was poor, with a median survival of 8 to 10 years [14,18]. With progress in surgery and medical technology, however, the 10-year survival rate is now about 78% to 91% [18,19]. Immunohistochemically, LAM cells usually express a representative smooth muscle phenotype and are consistently positive for HMB-45, a monoclonal melanoma-associated marker that reacts with a premelanosome-associated glycoprotein [20]. These cells can also be immunoreactive for estrogen and/or progesterone receptors [21].

However, more unusual cases have also been reported. A few cases of LAM have developed in postmenopausal women [19,22]. Many of these women were receiving or had received hormone replacement therapy. Several cases of LAM have even been reported in men since 1996 [23]. The retroperitoneum, mesentery, inguinal and supraclavicular lymph nodes, and ureters may also be sporadically involved. With respect to extrapulmonary locations, lymphadenopathy may be detected in the abdomen or pelvis, or chylous ascites, renal angiomyolipomas [24], uterine fibroid [25], or lymphaticoureteric and lymphaticovenous communications may develop [26,27]. Most cases of extrapulmonary LAM are accompanied by pulmonary involvement. Extrapulmonary LAM, especially retroperitoneal LAM, without lung involvement has rarely been reported. Kebria identified 9 cases of retroperitoneal LAM among 21 cases of extrapulmonary LAM, and Tanaka reported one case of retroperitoneal LAM in Japan. Almost all manifestations in these patients were acute abdominal pain or abdominal masses. To the best of our knowledge, the patient described in the present report is the first clinically asymptomatic patient to receive a diagnosis of primary retroperitoneal LAM without pulmonary involvement. Immunohistochemical studies confirmed that the LAM cells in this case were reactive to smooth muscle actin, F8-R-Ag, D2-40, and CD34; contrary to other cases, these LAM cells were negative for HMB-45 expression. This case represents a unique exception to the typical course of LAM and will help physicians to have a better understanding of LAM.

Because the etiology of LAM remains unknown, no standard effective treatment has been established. Multiple treatments have been applied with various effects. Treatment of pulmonary LAM is based on hormonal manipulations with intramuscular or oral medroxyprogesterone to prevent progressive lung destruction [28], and lung transplantation may be performed in advanced cases [26,29-31]. Moreover, thoracostomy or chemical pleurodesis is recommended for chylous effusion. The treatment of extrapulmonary LAM is not well defined. Paracentesis or peritoneovenous shunting is recommended for patients with chylous ascites. Hormonal manipulation, such as treatment with progesterone, oophorectomy, tamoxifen, or gonadotropin-releasing hormone agonists, has demonstrated mixed responses [32]. However, when managing retroperitoneal LAM involving no other sites, surgical resection is the most commonly used strategy and the only therapeutic option because of the uncertain behavior of this entity.

The diagnosis of LAM can be a dilemma because of the rarity of this disease, the lack of diagnostic suspicion in most cases, and the need to distinguish malignant lesions and lymphoma from other benign entities such as cysts. Such a diagnostic dilemma occasionally results in a delayed or missed diagnosis. Matsui *et al.* stated that many patients with extrapulmonary LAM develop pulmonary LAM within 1 to 2 years after the initial diagnosis [3].

Based on the present report and a review of the literature, LAM may originate from the retroperitoneum with no clinical symptoms and form a huge abdominal mass, as in the present case. LAM should be considered as a differential diagnosis of an abdominal space-occupying lesion, particularly in women of reproductive age, patients with tuberous sclerosis complex, or postmenopausal women who have undergone or are undergoing hormonal replacement therapy. The performance of high-resolution CT of the chest, abdomen, and pelvis in patients suspected to have LAM is strongly suggested because these imaging studies are noninvasive, sensitive, specific, and able to prevent a delayed or missed diagnosis and unnecessary surgical biopsy. Surgical resection is the first option to consider in patients with extrapulmonary LAM. Additionally, any patient diagnosed with retroperitoneal LAM must be studied and followed up with high-resolution chest CT to achieve an early diagnosis of possible pulmonary involvement, which will facilitate treatment in the early stages of the disease and improve the prognosis.

Further reports of LAM, clinical trials, and research will hopefully provide a better understanding of the etiology, diagnosis, and treatment options for patients affected by LAM.

## Conclusions

We herein describe a patient with PA-LAM with no respiratory or abdominal symptoms and an absence of any pulmonary imaging finding during a 6-month follow-up period. The diagnosis of PA-LAM was not made until a pathohistological examination was performed after surgical resection. This case showed that when a diagnosis of LAM is temporarily impossible, timely surgical treatment may be helpful for an early diagnosis, thereby improving the prognosis.

## Consent

Written informed consent was obtained from the patient for publication of this case report and any accompanying images. A copy of the written consent is available for review by the Editor-in-Chief of this journal.

## Abbreviations

CT: computed tomography
LAM: lymphangioleiomyomatosis
PA-LAM: primary abdominal LAM
US: ultrasonography

## References

[1] Maria Grazia F, Francesca S, Ivan L, Domenico P, Rosalia R (2005). Abdominal lymphangioleiomyomatosis in a man with Klinefelter syndrome: the first reported case. Ann Diagn Pathol.

[2] Calvo E, Amarillas L, Mateos MA, Orradre JL, Gilsanz G, Alvarez-Sala JL (1996). Lymphangioleiomyomatosis, chylous ascites, and diet. Dig Dis Sci.

[3] Matsui K, Tatsuguchi A, Valencia J, Yu Z, Bechtle J, Beasley MB (2000). Extrapulmonary lymphangioleiomyomatosis (LAM): clinicopathologic features in 22 cases. Hum Pathol.

[4] Chu SC, Horiba K, Usuki J, Avila NA, Chen CC, Travis WD (1999). Comprehensive evaluation of 35 patients with lymphangioleiomyomatosis. Chest.

[5] Johnson SR, Tattersfield AE (2000). Clinical experience of lymphangioleiomyomatosis in the UK. Thorax.

[6] Grzegorek I, Drozdz K, Podhorska-Okolow M, Szuba A, Dziegiel P (2013). LAM cells biology and lymphangioleiomyomatosis. Folia Histochem Cytobiol.

[7] Steagall WK, Taveira-DaSilva AM, Moss J (2005). Clinical and molecular insights into lymphangioleiomyomatosis. Sarcoidosis Vasc Diffuse Lung Dis.

[8] Lu HC, Wang J, Tsang YM, Lin MC, Li YW (2003). Lymphangioleiomyomatosis initially presenting with abdominal pain: a case report. Clin Imaging.

[9] Wong YY, Yeung TK, Chu WC (2003). Atypical presentation of lymphangioleiomyomatosis as acute abdomen: CT diagnosis. AJR Am J Roentgenol.

[10] Costello LC, Hartman TE, Ryu JH (2000). High frequency of pulmonary lymphangioleiomyomatosis in women with tuberous sclerosis complex. Mayo Clin Proc.

[11] Sun Y, Gallacchi D, Zhang EY, Reynolds SB, Robinson L, Malinowska IA (2014). Rapamycin-resistant PARP1 overexpression is a potential therapeutic target in lymphangioleiomyomatosis (LAM). Am J Respir Cell Mol Biol.

[12] Bissler JJ, McCormack FX, Young LR, Elwing JM, Chuck G, Leonard JM (2008). Sirolimus for angiomyolipoma in tuberous sclerosis complex or lymphangioleiomyomatosis. N Engl J Med.

[13] Jonson SR, Tattersfield AE (1999). Decline in lung function in lymphangioleiomyomatosis: relation to menopause and progesterone treatment. Am J Respir Crit Care Med.

[14] Urban T, Lazor R, Lacronique M, Murris M, Labrane S, Valeyre D (1999). Pulmonary lymphangioleiomyomatosis: a study of 69 patients. Medicine.

[15] Ye L, Jin M, Bai C (2010). Clinical analysis of patients with pulmonary lymphangioleiomyomatosis (PLAM) in mainland China. Respir Med.

[16] Logginidou H, Ao X, Russo I, Henske EP (2000). Frequent estrogen and progesterone receptor immunoreactivity in renal angiomyolipomas from women with pulmonary lymphangioleiomyomatosis. Chest.

[17] Sherrier RH, Chiles C, Roggli V (1989). Pulmonary lymphangioleiomyomatosis CT findings. Am J Radiol.

[18] Hayashida M, Seyama K, Inoue Y, Fujimoto K, Kubo K (2007). The epidemiology of lymphangioleiomyomatosis in Japan: a nationwide cross-sectional study of presenting features and prognostic factors. Respirology.

[19] Kitaichi M, Nishimura K, Itoh H, Izumi T (1995). Pulmonary lymphangioleiomyomatosis: a report of 46 patients including a clinicopathologic study of prognostic factors. Am J Respir Crit Care Med.

[20] Lack EE, Dolan MF, Finisio J, Grover G, Singh M, Triche TJ (1986). Pulmonary and extrapulmonary lymphangioleiomyomatosis. Report of a case with bilateral renal angiomyolipomas, multifocal lymphangioleiomyomatosis, and a glial polyp of the endocervix. Amer J Surg Path.

[21] Berger U, Khaghani A, Pomerance A, Yacoub MH, Coombes RC (1990). Pulmonary lymphangioleiomyomatosis and steroid receptors. An immunocytochemical study. Am J Clin Pathol.

[22] Sinclair W, Wright JL, Churg A (1985). Lymphangioleiomyomatosis presenting in a postmenopausal woman. Thorax.

[23] Korobowicz E, Sierocińska-Sawa J (1996). Pulmonary lymphangioleiomyomatosis - a case report. Pol J Pathol.

[24] Sullivan EJ (1998). Lymphangioleiomyomatosis: a review. Chest.

[25] Wahedna I, Cooper S, Williams J, Paterson IC, Britton JR, Tattersfield AE (1994). Relation of pulmonary lymphangioleiomyomatosis to use of the oral contraceptive pill and fertility in the UK: a national case control study. Thorax.

[26] Carrington CB, Cugell DW, Gaensler EA, Marks A, Redding RA, Russi EW (1977). Lymphangioleiomyomatosis: physiologic-pathologic-radiologic correlations. Am Rev Respir Dis.

[27] Rumancik WM, Bosniak MA, Rosen RJ, Hulnick D (1984). Atypical renal and pararenal hamartomas associated with lymphangiomyomatosis. AJR.

[28] Svendsen TL, Viskum K, Hansborg N, Thorpe SM, Nielsen NC (1984). Pulmonary lymphangioleiomyomatosis: a case of progesterone receptor positive lymphangioleiomyomatosis treated with medroxyprogesterone, oophorectomy and tamoxifen. Br J Dis Chest.

[29] Wolff M (1973). Lymphangiomyoma: clinicopathologic study and ultrastructural confirmation of its histogenesis. Cancer.

[30] Banner AS, Carrington CB, Emory WB, Kittle F, Leonard G, Ringus J (1981). Efficacy of oophorectomy in lymphangioleiomyomatosis and benign metastasizing leiomyoma. N Engl J Med.

[31] McCarthy KS, Mossler JA, McLelland R, Sieker HO (1980). Pulmonary lymphangiomyomatosis responsive to progesterone. N Engl J Med.

[32] Kelly J, Moss J (2001). Lymphangioleiomyomatosis. Am J Med Sci.

